# Treatment of 3D In Vitro Tumoroids of Ovarian Cancer Using Photochemical Internalisation as a Drug Delivery Method

**DOI:** 10.3390/biomedicines11020572

**Published:** 2023-02-15

**Authors:** Layla Mohammad Hadi, Katerina Stamati, Elnaz Yaghini, Alexander J. MacRobert, Marilena Loizidou

**Affiliations:** Division of Surgery & Interventional Science, University College London, London NW3 2QG, UK

**Keywords:** ovarian cancer, photochemical internalisation, photodynamic therapy, tumouroids, 3D compressed collagen construct, hypoxia

## Abstract

Photochemical internalisation (PCI) is a means of achieving spatio-temporal control of cytosolic drug delivery using sub-lethal photodynamic therapy (PDT), with a photosensitiser that can be activated by non-ionising visible light. Various 3D models including those developed at our laboratory, where spheroids are grown in a compressed collagen matrix, have been used for studying anti-cancer drug effects. However, the use of a more biomimetic tumouroid model which consists of a relatively hypoxic central cancer mass surrounded by its microenvironment (stroma) has not yet been explored in either toxicity or phototoxicity studies involving PCI. Here, we examined the efficacy of PCI using a porphyrin photosensitiser and a cytotoxin (Saporin) on ovarian cancer tumouroids, with HEY ovarian cancer cells in the central cancer compartment, and HDF fibroblast cells and HUVEC endothelial cells in the surrounding stromal compartment. The efficacy was compared to tumouroids treated with either Saporin or PDT alone, or no treatment. PCI treatment was shown to be effective in the tumouroids (determined through viability assays and imaging) and caused a considerable decrease in the viability of cancer cells both within the central cancer mass and those which had migrated into the stroma, as well as a reduction in the cell density of surrounding HUVEC and HDFs. Post-treatment, the mean distance of stromal invasion by cancer cells from the original cancer mass following treatment with Saporin alone was 730 μm vs. 125 μm for PCI. PDT was also effective at reducing viability in the central cancer mass and stroma but required a higher photosensitiser dose and light dose than PCI. Tumouroids, as tissue mimics, are suitable models for interrogating multicellular events following pharmacological assault.

## 1. Introduction

Photochemical internalisation (PCI) utilises sub-lethal photodynamic therapy (PDT) to deliver macromolecular toxins or anti-cancer drugs to the cytosol following endocytic uptake and entrapment, thereby reducing the risk of endolysosomal degradation, and enabling migration to their intracellular target sites of action [1,2]. This is achieved through the co-administration of a photosensitiser that localises in endolysosomal membranes, which then enables photo-induced permeabilisation of the membranes and release of the entrapped agents into the cytosol. The effectiveness of PCI in treating cancer has been demonstrated in numerous in vitro, in vivo and clinical studies [3,4,5,6,7,8,9]. The utility of 3D models is well-proven for PDT [10] but there have comparatively been few studies investigating their use for PCI. Since PDT and PCI are both oxygen-dependent treatments, 3D model studies offer a key advantage over conventional monolayer studies since the rate of oxygen diffusion is restricted by the extracellular matrix or cell density in a spheroid.

In our previous studies, PCI proved effective in treating simple in vitro 3D compressed cancer masses, which are non-spheroid and spheroid constructs of ovarian cancer in compressed collagen, using TPPS_2a_ as the photosensitiser and Saporin as the anti-cancer drug, compared to exposing the constructs to PDT or Saporin alone [11,12]. Under hypoxic conditions, however, both PDT and PCI were less effective. In the current study, we have advanced our research by investigating whether the same increased therapeutic efficiency can be observed when combining TPPS_2a_ and Saporin to treat complex, more biomimetic tumouroids compared to applying each drug separately.

Complex tumouroids mimic the heterogeneity of solid cancer tissue, by including both cancer masses and the surrounding stroma. Of the major types of cells found in the microenvironment [13], we incorporated fibroblasts and endothelial cells. Tumoroids allow the interactions between cancer cells and the surrounding stroma to be studied as the cancer progresses and metastasises. Since such models recapitulate the in vivo scenario, the impact of the environment on drug uptake and effectiveness can also be determined more accurately than in 2D and simple 3D cancer models [13,14,15,16,17]. Furthermore, the compression of the tumouroid and the removal of the interstitial fluid creates a far denser extracellular matrix environment (5000 KPa) hich is similar to human tumours. Considering that PCI and PDT both consume molecular oxygen, and therefore, rely on oxygen transport to cells, the matrix density is a relevant factor since a higher density reduces the rate of oxygen diffusion, and therefore, the replenishment of the oxygen supply to cells in the central cancer mass. Moreover, in previous studies on the same type of two-compartment construct but using colorectal cancer cells (HT29), oxygenation levels in the central cancer mass were found to be hypoxic (<1 mmHg) compared to the surrounding stroma owing to the denser cell population in the central cancer mass [18]. This feature is also relevant to PCI and PDT which are aimed at treating solid tumours that are often hypoxic at their core. We have previously shown that the efficacy of both PCI and PDT are diminished under hypoxic conditions [8].

## 2. Materials and Methods

### 2.1. Cell Maintenance

Human ovarian carcinoma cell line (HEY), (gifted by the UCL Cancer institute), Human dermal fibroblasts (HDFs) and Human umbilical vein endothelial cells (HUVECs) (stocks in the UCL Division of Surgery and interventional Sciences) were cultured under routine conditions in Dulbecco’s modified Eagle medium (DMEM/F-12, low glucose, Sigma Aldrich, Dorset, UK), High glucose DMEM (Thermo Fisher Scientific, Hemel Hempstead, UK) and Endothelial cell growth medium with supplements (PromoCell), respectively. The cell culture media were supplemented with 10% heat-inactivated foetal bovine serum (FBS, Thermo Fisher Scientific, Hemel Hempstead, UK) and 1% penicillin (5000 units/mL) and streptomycin (5000 μg/mL) (Thermo Fisher Scientific, Hemel Hempstead, UK).

### 2.2. Tumouroid Manufacture

**Figure 1:**
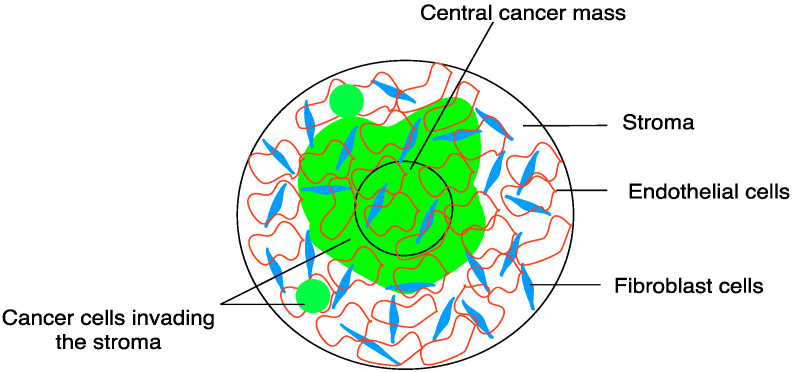
Schematic of ovarian tumouroid. The central cancer mass is manufactured by mixing HEY ovarian cancer cells and collagen type 1, allowing it to set as hydrogel and applying absorbers (RAFT^TM^) to remove liquid and create a stiff, compressed 3D tissue. The cancer mass is placed in a large “stromal” hydrogel populated by fibroblasts (HDFs) and endothelial cells (HUVECs), with further addition of stromal hydrogel-cell mix. The whole construct is compressed (RAFT^TM^) to produce complex tumouroids. Tumouroids are allowed to mature for 7 days, with cancer invading from the cancer mass into the stroma, prior to exposure to the drug.

Tumouroids were created using a method modified from [14]. Briefly, the hydrogels were prepared from a mixture containing 10× MEM (used as colour indicator) (10% of total volume), Rat Tail Collagen Type I (First Link UK Ltd. Custom Bio-Reagents, Bimingham, UK) (80% of total volume) before undergoing neutralisation using neutralising solution (5.8% of total volume) made from 1.65 M NaOH and 840 mM HEPES buffer solution (Thermo Fisher Scientific, Hemel Hempstead, UK); 3D compressed cancer masses, (4.2% of total volume) were prepared initially by seeding 50,000 HEY cells (based on previous optimisation) [11] at volumes of 240 μL per well in a 96 well plate (TPP, Scientific Laboratory Supplies, Nottingham, UK). The cancer-collagen mixtures were incubated at 37 °C for 15 min to set before being compressed using absorbers (Lonza, Slough, UK) at room temperature for a further 15 min. After the removal of the absorbers, fresh media was added to each 3D compressed cancer mass followed by a short incubation at 37 °C while the stromal component was manufactured. The stroma was created following the same steps as above to prepare hydrogels seeded with a mixture of HDFs and HUVECs at densities of 25,000 and 50,000 cells, respectively [14]. First, half of the total volume per well (650 μL) of the stroma cell/collagen mix was added to each well in a 24-well plate (Corning Costar, Fisher Scientific, Loughborough, UK) and allowed to set slightly at room temperature for 5 min; 3D compressed cancer masses were then removed from the 96 well plates and each placed on top of a slightly set stroma hydrogel in the 24 well plates, and topped with an additional 650 μL of the stroma cell/collagen mix (totalling 1.3 mL per well) and incubated at 37 °C for 15 min to set completely. At that time, the complex tumouroid hydrogels underwent compression using absorbers. Upon removal of the absorbers, 200 μL of each media (600 μL in total) appropriate to the individual cell lines incorporated in the tumouroids was added per well. Tumouroids with acellular stroma termed simple tumouroids were also produced. For simple tumouroids, media was added to the collagen mix instead of the stromal cell mix before the compressed cancer mass was sandwiched between two layers of the acellular stroma. The simple tumouroids were then left to set before undergoing compression in the same way as complex tumouroids. They were then topped with 600 μL of low glucose DMEM. Based on previous experiments [11], the tumouroids were incubated at 37 °C for 7 days to allow the HEY cells to form spheroid-like structures and invade the stroma prior to undergoing treatment. Half of the cell culture media in each well was changed daily to ensure that the cells received enough nutrients. For specific experiments, 3D compressed collagen constructs were created using only HDFs (25,000 cells/construct). Figure 1 is a schematic illustration of a complex ovarian tumouroid.

### 2.3. PDT/PCI Phototoxicity Studies in Ovarian Tumouroids

**Figure 2:**
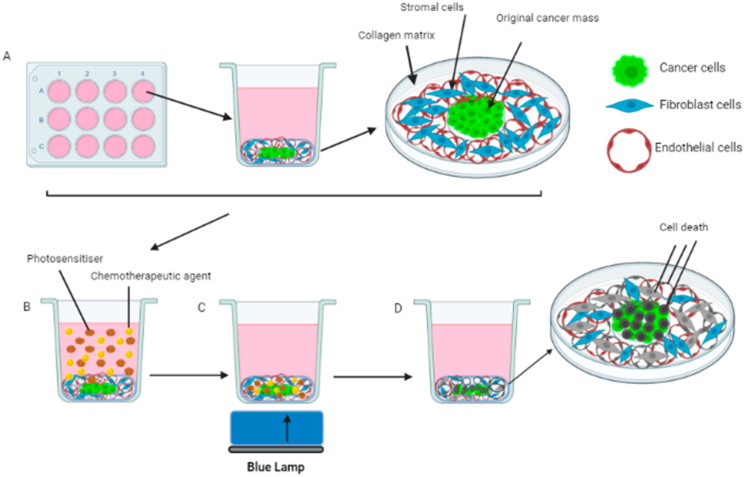
Fabrication of tumouroid constructs. (**A**) Complex tumouroids are constructed in well plates; (**B**) Addition of photosensitiser (TPPS_2a_) and chemotherapeutic agent (Saporin) to the complex tumouroid for cell uptake followed by washing; (**C**) Irradiation of tumouroid with blue light and generation of ROS; (**D**) Dead cancer cells are formed in the tumouroid.

The addition of drugs to tumouroids was carried out on day 7 post-manufacture and experiments were concluded by day 10. The concentrations of TPPS_2a_ (Frontier Scientific) used for the PDT treatment were 0.5 μg/mL and 1μ g/mL. For PCI treatment the concentration 0.5 μg/mL was used based on previous experiments with 3D compressed cancer masses which indicated that this dose caused sub-lethal PDT effects. In the PCI experiment, the tumouroids were either incubated with TPPS_2a_ only, Saporin (Sigma Aldrich, Dorset, UK) only (10 nM and 20 nM selected due to effectiveness demonstrated in previous PCI experiments on 3D compressed cancer masses) and combinations of TPPS_2a_ and Saporin for each Saporin concentration. The tumouroids were then incubated for 20 h at 37 °C before the drug was removed. Tumouroids were washed with phosphate-buffered saline (PBS, PH 7.4, Sigma Aldrich, Dorset, UK) and replaced with fresh media. Incubation at 37 °C was repeated for a further 4 h followed by light irradiation treatment using a blue lamp (Lumisource, 475 nm, 7 mW/cm^2^, PCI Biotech, Oslo, Norway) for periods of 3 min (for PDT and PCI) or 5 min (for PDT). The tumouroids were again incubated at 37 °C for a further 48 h before termination. A number of investigations were undertaken, including imaging and viability, as described below. Control groups with no drugs added and with or without light exposure were also assessed. Various experiments were conducted using simple and complex tumouroids as well as 3D compressed collagen constructs and these are indicated in the results. Figure 2 is a schematic illustration of the PCI treatment of a complex tumouroid.

### 2.4. Immunostaining for Fluorescence Imaging

Following the termination of the experiment, complex tumouroids were fixed using Neutral Buffered Formalin (10%) (Sigma Aldrich, Doset, UK) for 10 min and then washed with PBS. The cells within tumouroids were permeabilised using 1% BSA and 0.3% Triton X solution for 30 min before they were washed with PBS once again. The tumouroids were stained using Anti-Cytokeratin 7 (Alexa Fluor 488) λ_Ex/Em_: 490/525 conjugated antibody (Abcam, Cambridge, UK) (for HEY cells), Anti-Vimentin (Alexa Fluor 405) λ_Ex/Em_: 401/421 conjugated antibody (Abcam, Cambridge, UK) (for HDFs) and Anti-CD31 (PerCP-eFluor 710) λ_Ex/Em_: 482/709 conjugated antibody (eBioscience, Thermo Fisher Scientific, Hemel Hempstead, UK) (for HUVECs). The anti-cytokeratin 7 (Alexa Fluor 488) and anti-CD31 (PerCP-eFluor 710) antibodies were mixed in 1% BSA and 0.3% Triton X solution at ratios of 1/100 and 1/500, respectively, before they were added to the tumouroids and incubated at 4 °C overnight for 20 h. The antibody solution was removed, and tumouroids were washed three times with PBS each time incubating at room temperature for 5 min. Finally, the anti-vimentin (Alexa Fluor 405) conjugated antibody stain was diluted in PBS at a ratio of 1/200, added to the tumouroids and incubated for 3 h at room temperature. At that time, tumouroids were washed with PBS (three times, 10 min each) and placed between a glass slide and cover slip in preparation for imaging. Images were taken with an Olympus fluorescence microscope (Olympus BX63, Olympus UK, Southend-on-Sea, UK) using channels FITC (for HEY cells), DAPI (for HDFs) and CY7 (for HUVECs) as well as 4× and 10× objectives.

### 2.5. Tumouroid Re-Growth Studies

Simple tumouroids were treated with PDT using TPPS_2a_ (0.5 μg/mL) and a 3 min illumination period, 7 days post-manufacture. The tumouroids were then incubated at 37 °C for a further 7 days before terminating, staining with Cytokeratin 7 (Alexa Fluor 488) conjugated antibody and imaging using an Olympus fluorescence microscope (as above).

### 2.6. Cell Viability Assay

The viability of simple tumouroids was determined using the Alamar Blue assay (Invitrogen, Thermo Fisher Scientific, Hemel Hempstead, UK). Briefly, 48 h after light illumination, Alamar Blue dye solution (10% of the total volume in culture medium) was added to the wells and incubated at 37 °C for 4 h. Afterwards, the supernatant from each well was transferred into black well plates. Fluorescence was measured following excitation at 530 nm and emission was detected at 620 nm using a fluorescence plate reader (Fluoroskan Ascent, Thermo Scientific, Waltham, MA, USA).

### 2.7. Image Analysis

The images were analysed using open-source Image J software to measure the distance within the stroma that was invaded by cancer cells in simple tumouroids post-PDT and PCI treatment. For each tumouroid, eight measurements were taken from the edge of the original cancer mass to the farthest cancer cell cluster within the stroma in a clockwise manner. Each condition had three tumouroid repeats, thereby generating 24 measurements per condition from which averages were taken.

### 2.8. Statistical Analysis

Results were analysed using 2-way ANOVA with posthoc analysis and shown as means ± standard deviation (SD). Values of *p* < 0.05 were considered statistically significant.

## 3. Results

### 3.1. Growth and Stromal Invasion in Complex Tumouroids

**Figure 3:**
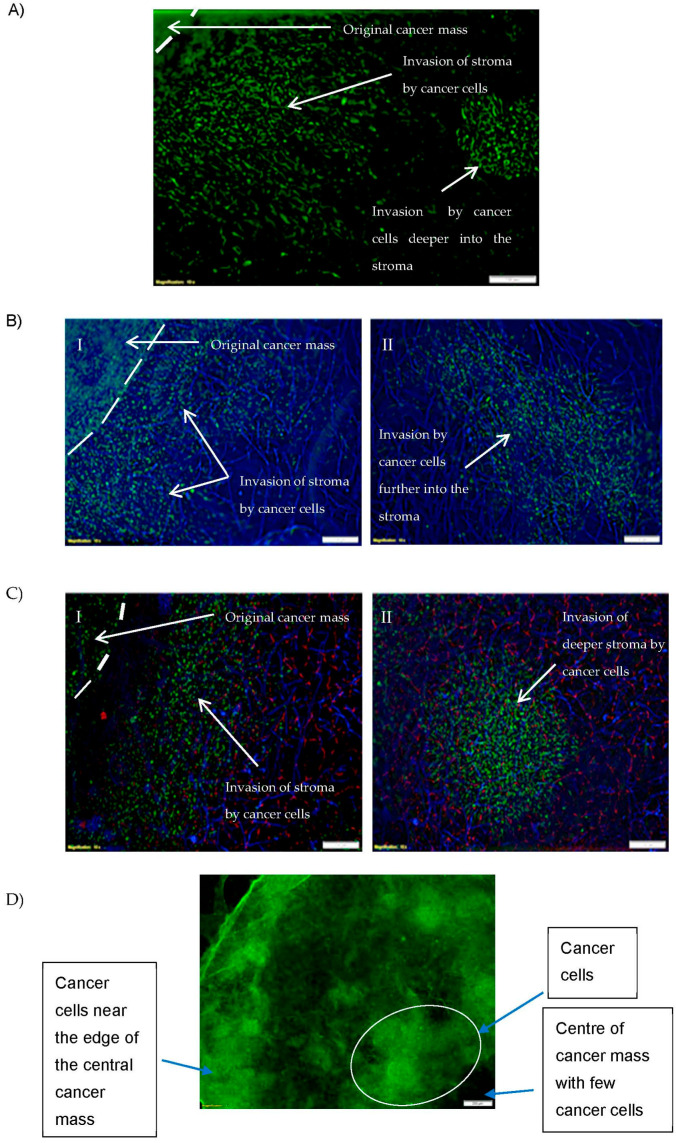
Cancer cell growth and stroma invasion by cancer cells in 3D ovarian tumouroids. (**A**) Invasion by cancer cells (HEY) from the original cancer mass into the adjacent stroma and deeper stroma (a-cellular). (**B,C**) Invasion by cancer cells (HEY) from the original cancer mass into stroma with fibroblast (HDF) and endothelial cells (HUVEC). (**BI**,**CI**) Invasion of stroma by cancer cells near the original cancer mass. (**BII**,**CII**) Invasion of deeper stroma by cancer cells. (**D**) Distribution of HEY cells within the central cancer mass. Cancer, fibroblast and endothelial cells were stained with anti-cytokeratin 7 (Alexa Fluor 488) λ_Ex/Em_: 490/525 (green), anti-Vimentin (Alexa Fluor 405) λ_Ex/Em_: 401/421 (blue) and anti-CD31 (PerCP-eFluor 710) λ_Ex/Em_: 482/709 (red) conjugated antibodies, respectively. The scale bar presented is 100 μm for (**A**–**C**) and 200 μm for (**D**).

By day 10 the cancer cells had proliferated and invaded the stroma both in the area adjacent to the central cancer mass and further into the stroma. As shown in Figure 3, the cancer cells were able to invade the stroma with the stromal fibroblasts and endothelial cells present (Figure 3B,C) as effectively as they could invade the acellular stroma (Figure 3A). Figure 3(BI,BII) shows a tumouroid consisting of all three cell lines, only the stained cancer and fibroblast cells has been presented. Interestingly most of the cancer cells grew mainly at the outer edges of the central cancer compartment (Figure 3D).

### 3.2. PDT and PCI in Tumouroids

**Figure 4:**
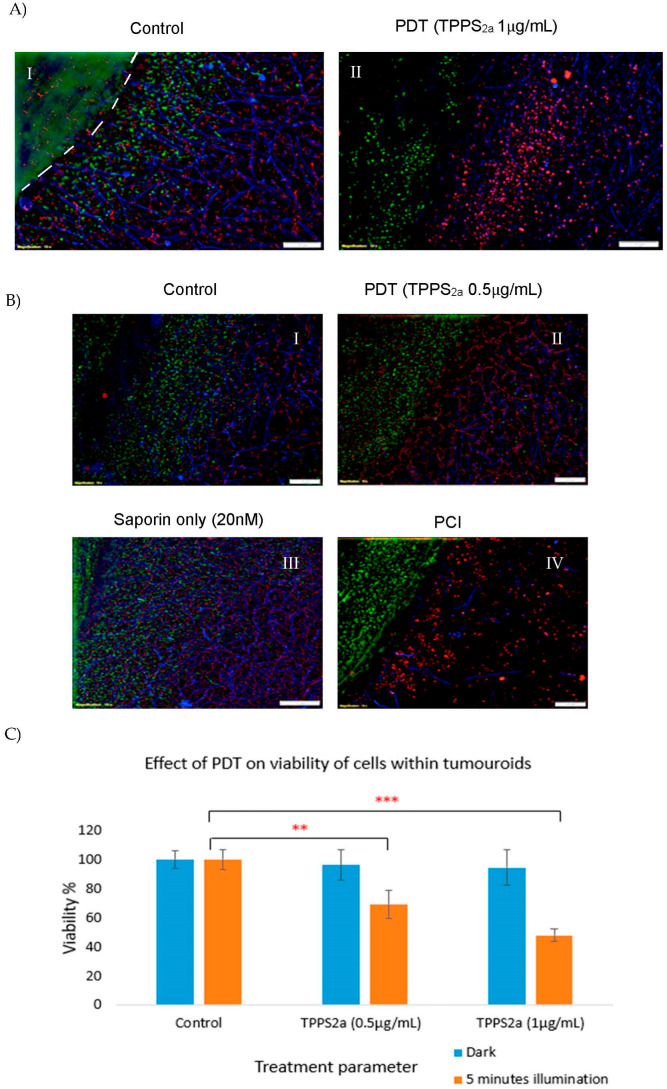
PDT and PCI treatment in 3D complex tumouroids. (**A**) Control complex tumouroid construct (**I**), and PDT in complex tumouroids using TPPS_2a_ (1 μg/mL) and 5 min of illumination (**II**); (**B**) Control complex tumouroid construct (**I**), PDT in complex tumouroid constructs using TPPS_2a_ (0.5 μg/mL) only (**II**), Saporin (20 nM) only treatment (**III**) or a combination of both drugs (PCI) (**IV**) and 3 min of light exposure. Cancer, fibroblast and endothelial cells were stained with anti-cytokaretin 7 (Alexa Fluor 488) λ_Ex/Em_: 482/709 (red) conjugated antibodies, respectively. The scale bar presented is 100 μm for Figure 4A,B; (**C**) Overall percentage viability of cells in simple tumouroids after treatment with PDT using TPPS2a 0.5 μg/mL and 1 μg/mL and Alamar blue assay at 48 h post illumination with blue lamp. ** *p* < 0.01 and *** *p* < 0.001. These *p*-values show the significant difference between PDT-treated and untreated controls.

PCI treatment using TPPS_2a_ (0.5 μg/mL) and Saporin (20 nM) and a 3 min illumination period was carried out on complex tumouroids (Figure 4B). PDT-only treatment (Figure 4(BII)) caused a reduction in cancer cells invading the stroma and a reduction in stromal fibroblasts compared to the control and Saporin-only treated tumouroids (Figure 4(BI,BIII)); however, the endothelial cells were not significantly affected. Photosensitiser administration alone without light produced no significant difference in viability. Furthermore, the quantitative data from the Alamar blue experiments showed that overall, the tumouroids with acellular stroma which were treated with TPPS_2a_ (1 μg/mL) had significantly lower percentage viability than those treated with TPPS_2a_ (0.5 μg/mL) and their control counterparts (Figure 4C). In the PCI treated tumouroids further destruction of the cancer cells in the central cancer mass and those invading the stroma was observed in addition to the damage to stromal endothelial and fibroblast cells (Figure 4(BIV)).

The complex tumouroids were also exposed to PDT treatment using TPPS_2a_ (1 μg/mL) and a 5 min illumination period to study the effect of a stronger PDT treatment on the cancer mass and the stroma (Figure 4A). Compared to the controls (Figure 4(AI)) which showed invasion of cancer cells from the central cancer mass into the stroma, and healthy-looking stromal cells, the PDT-treated tumouroids (Figure 4(AII)) presented a different picture with fewer cells observed in the central cancer mass and reduced invasion of cancer cells into the stroma. In the stroma, there were fewer fibroblasts and the endothelial cells were disrupted.

**Figure 5:**
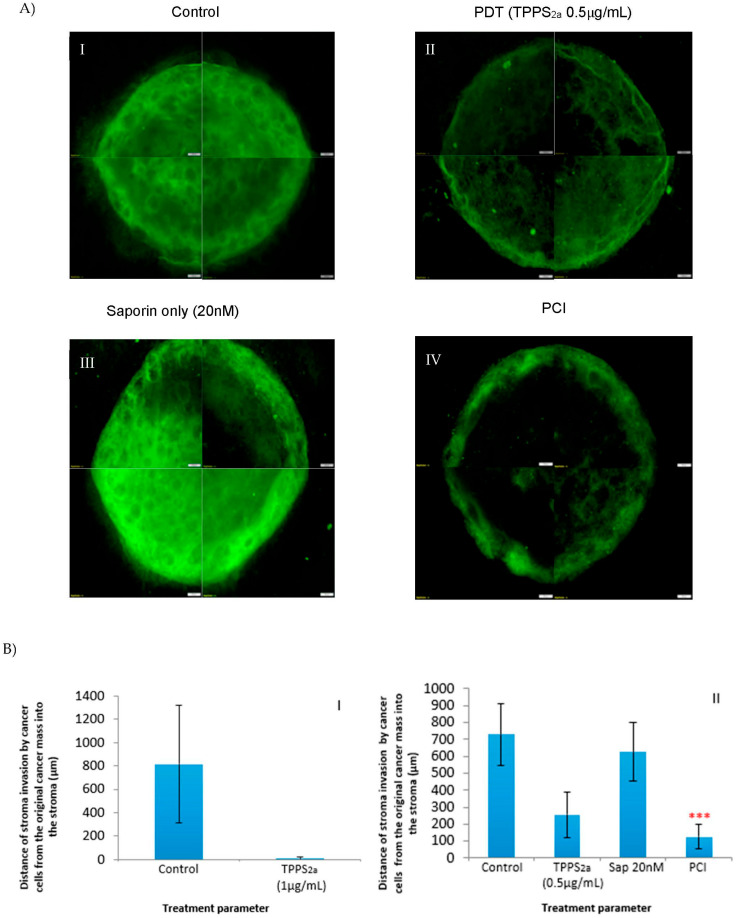
(**A**) Overall images of the cancer cell population stained with anti-cytokeratin 7 (Alexa Fluor 488) λEx/Em: 490/525 (green) within the central cancer mass of the simple tumouroids for each treatment condition; Control simple tumouroid construct (**I**), PDT in simple tumouroid construct using TPPS2a (0.5 μg/mL) only (**II**), Saporin only (**III**), and PCI (**IV**); (**B**) Histograms showing distance (μ M) invaded within the tumouroid stroma by cancer cells post PDT and PCI treatments described in Figure 4A and Figure 4B. Distance (μM) invaded within stroma invaded by cancer cells after PDT treatment using TPPS_2a_ (1 μg/mL) (**I**). Distance μ(M) invaded within stroma invaded by cancer cells after PCI treatment using TPPS_2a_ (0.5 μg/mL) and Saporin (20 nM) (**II**). The invasion distances presented are averages from 24 measurements taken from different areas all around the original cancer mass of each tumouroid. Each measurement was taken from the border of the original cancer mass to the furthest point of invasion in the stroma which lay in the same direction as the starting point of the measurement. *** *p* < 0.001 shows the significant difference in the distance invaded in the stroma by cancer cells post PDT and PCI. The scale bar presented in the images is 200 μm.

Interestingly the cancer cells showed a tendency to migrate to the border of the central cancer mass which, therefore, resulted in higher destruction of cells in the center of the cancer mass than those in the edges of the cancer mass after PDT and PCI treatments (Figure 5(AI–IV)).

The distance of cancer cell invasion into the stroma was measured in simple tumouroids treated with PDT (conditions presented in Figure 4A) and PCI (conditions presented in Figure 4B) to generate histograms shown in Figure 5B. An average was taken from 24 areas (three repeats, eight areas each) for each condition. As histogram 5BI shows, the cancer cells invaded far into the stroma in the control samples, but such an invasion is almost non-existent in the PDT-treated samples. Graph 5BII also shows a similar pattern to that observed in Figure 4B with a considerable invasion of the stroma by cancer cells in the control and Saporin only tumouroids, but much less in the PDT and PCI-treated samples, with a stronger effect being observed in the PCI group.

**Figure 6:**
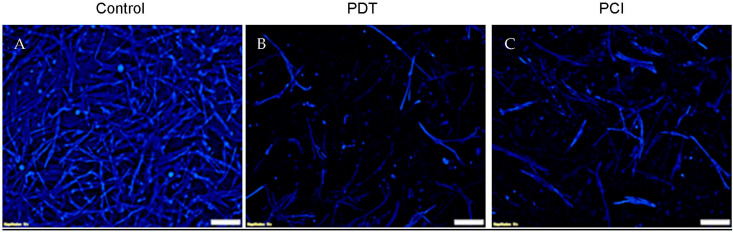
PDT and PCI treatment of fibroblast cells grown in 3D compressed collagen constructs using 3 min light illumination. (**A**) Control (**B**) PDT treatment using TPPS_2a_ (0.5 μg/mL); (**C**) PCI treatment using combination of TPPS_2a_ (0.5 μg/mL) and Saporin (20 nM). The images were obtained 48 h post-light illumination. The HDFs were stained with anti-Vimentin (Alexa Fluor 405) λ_Ex/Em_: 401/421 (blue). The scale bar presented is 100 μm.

To confirm the effect of the treatments on the fibroblasts, a further experiment was carried out where fibroblast-populated 3D compressed collagen constructs were created and exposed to PDT and PCI treatments (Figure 6). As Figure 6B,C show, both PDT and PCI have a similar negative effect on fibroblasts.

### 3.3. Tumour Regrowth after PDT

**Figure 7:**
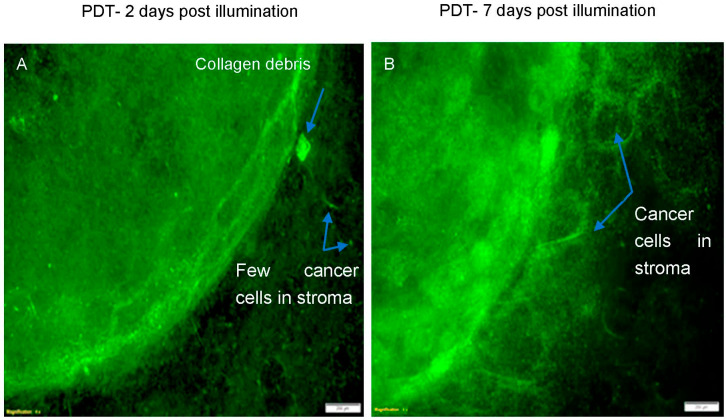
Regrowth of cancer cells in PDT-treated simple tumouroid 7 days post-treatment. The tumouroid was treated with TPPS_2a_ (0.5 μg/mL) and 3 min of light illumination. (**A**) Image of a tumouroid 2 days post light illumination; (**B**) Image of a tumouroid 7 days post light illumination. The cells were stained with anti-cytokeratin 7 (Alexa Fluor 488) λ_Ex/Em_: 490/525 (green) conjugated antibody. The scale bar presented is 200 μm.

**Figure 8:**
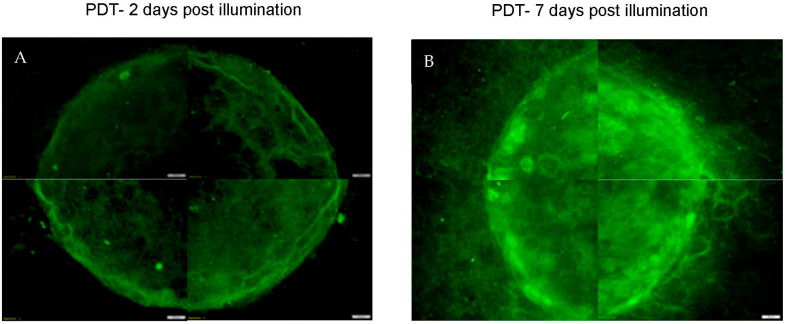
Regrowth of cancer cells in PDT-treated simple tumouroids 7 days post-treatment. The tumouroid was treated with TPPS_2a_ (0.5 μg/mL) and 3 min of light illumination. (**A**) Full image of a tumouroid 2 days post light illumination; (**B**) Full image of a tumouroid 7 days post light illumination. The cells were stained with anti-cytokeratin 7 (Alexa Fluor 488) λ_Ex/Em_: 490/525 (green) conjugated antibody. The scale bar presented is 200 μm.

In a further experiment, the rate of cancer cell regrowth post-PDT was examined where the simple tumouroids were incubated for a further 7 days after treatment. Figure 7 shows an example of a tumouroid that had undergone PDT using TPPS_2a_ (0.5 μg/mL) followed by 3 min of light illumination and allowed to recover for 7 days prior to fixing and staining. Overall images of the treated tumouroids are shown in Figure 8. In comparison to tumouroids treated in the same way, but fixed and stained 2 days (48 h) post-treatment, the recovered tumouroids exhibited a denser population of cancer cells in the central cancer mass as well as a considerable invasion of the cancer cells into the stroma (Figure 7B and Figure 8B).

## 4. Discussion

Ovarian cancer is one the deadliest cancers affecting women worldwide and often metastasises to the peritoneum causing the development of ascites. Treatment of ovarian cancer can be difficult as it is usually diagnosed late. The light-based treatments, PDT and PCI, have the potential to offer a non-invasive treatment for peritoneal carcinomatosis through the illumination of the peritoneal cavity. In this study, we used an in vitro 3D multicellular tumouroid model, to study the effect of PDT/PCI on ovarian cancer cells surrounded by a tumour microenvironment containing stromal cells, namely fibroblasts and endothelial cells. The tumouroid is constructed using collagen as the basic matrix and compressed to mimic the stiffness of native cancer tissues. Fluorescence imaging was used (anti-Cytokeratin 7/Alexa Fluor 488) to locate cancer (HEY) cells, anti-Vimentin (Alexa Fluor 405) to locate fibroblast (HDF) cells, and anti-CD31 (PerCP-eFluor 710) to stain platelet-endothelial cell adhesion molecule-1 expressed on the surface of endothelial (HUVEC) cells.

The simple tumouroids or two-compartment tumouroids without stromal cells enabled precise measurements of the viability of the cancer cells to be made after treatment. The complex two-compartment tumouroid with stromal cells enabled us to investigate the impact of PDT/PCI on both cancer cells and stromal cells stemming from the aforementioned advantage of being able to image the stromal cells clearly since they were seeded in the compartment surrounding the central cancer mass. In a previous study from our laboratory, we demonstrated the feasibility of detecting the impact of a tyrosine kinase inhibitor, Pazopanib, on renal cancer cells and endothelial cells using the complex tumouroid model [19]. In that study, it was shown that the inhibitor affected cancer growth, invasion and stromal morphology. In the initial experiments of our current study, we wanted to know how cancer cell growth and stromal invasion were affected by the presence of stromal cells. In that experiment, it was found that the cancer cells were able to grow and invade the further areas of the stroma regardless of the presence or absence of stromal cells. Therefore, we were able to use simple tumouroids to measure the viability of cancer cells within tumouroids and the experiment explored the effects of PDT and PCI on cancer cells that had invaded the stroma. However, to investigate the impact of PDT/PCI on cancer and stromal cells, complex tumouroids were used. The model is particularly suitable for studies of PCI and PDT, which are oxygen-dependent treatments, since the central cancer mass is relatively hypoxic thereby mimicking the hypoxic cores of solid tumours, and the near-physiological collagen density of the surrounding stroma reduces the rate of oxygen diffusion by an order of magnitude compared to uncompressed collagen hydrogels [20]. Interestingly, in our studies on simple tumouroids, we observed that in comparison to the control and saporin only tumouroids, PDT and PCI treatments using TPP_2a_ (0.5 µg/mL) and saporin (20 nM), were able to cause a reduction in the cancer cell population within the original mass with PCI exerting a stronger effect (Figure 5A).

Magdeldin et al. (2017) [14] used a similar 3D culture system to develop a colorectal tumouroid model using HT29 and HCT116 (colorectal cancer cells), HDFs (fibroblast cells) and HUVECS (endothelial cells) to study the mechanisms involved in cancer progression. Two different mechanisms of migration were identified which showed that generally, the HT29 cells invaded the stroma as cellular aggregates whereas the HCT116 cells, a more aggressive cell line, invaded as epithelial cell sheets. In comparison, in our study, the cancer cells invaded the stroma initially as epithelial cell sheets but the cancer cells migrated further into the stromal environment and invaded as aggregates.

Other methods have also been used for making complex in vitro 3D models. For example, Jaganathan et al. (2014) [21] developed a co-culture breast cancer model consisting of breast cancer cells SUM159 and MDA-MB-231 as well as fibroblast cells Hs785bst, 293T and patient-derived cancer-associated fibroblasts to demonstrate the influences of the tumour microenvironment on drug efficiency. For this study, different combinations of breast cancer and fibroblast cells were co-cultured at different ratios in ultra-low attachment well plates before being subjected to magnetic levitation.

Amann et al. (2017) [22] on the other hand used the hanging drop technology to develop a tri-culture model consisting of non-small cell lung cancer (NSCLC) cell lines (A549 and Colo699) as well as a fibroblast cell line (SV 80) and two different endothelial cell lines (CC-2527 and CC-2935) to study the effect of anti-angiogenic drugs. Endothelial cells formed small colonies in microtissues containing Colo699 cells, and tube-like structures mostly in the stromal area of microtissues containing A549, suggesting that there are cancer cell line–specific cross-talk events. Furthermore, the use of antiangiogenic drugs, such as bevacizumab and nindetanib, resulted in a significant reduction in the migration of endothelial cells into microtissues.

Our tumouroid model offers several advantages over many other 3D culture systems used. One of the benefits of using the two-compartment tumouroid models is that the stromal cells are grown in the surrounding compartment and it is, therefore, much easier to image the stromal cells compared to a 3D model with spheroids and stromal cells all in the same matrix compartment. Other advantages associated with the use of the tumouroid system include the tuneable nature of the model which enables control of the matrix density up to physiological ECM densities thus better mimicking an in vivo biological environment. However, there are some drawbacks to the use of the tumouroid system, in particular its manual preparation, although this could partly be automated in the future.

We found that the PDT-only treatment using TPPS_2a_ (0.5 μg/mL) and 3 min of exposure to light caused a reduction in the apparent population of cancer cells in the central cancer mass and those invading the stroma, as well as fibroblasts and endothelial cells compared to the control and Saporin only treated tumoroids (Figure 4B). In contrast, the PCI treatment of tumouroids elicited further destruction of the central cancer mass and a reduction in stromal invasion in addition to the damage to endothelial and fibroblast cells resident within the stroma (Figure 4B). Therefore, when using equivalent amounts of photosensitiser and the same light exposure, we demonstrated that PCI was more effective than PDT. Since the treatment is oxygen dependent and the availability of oxygen in the tumoroids can be limited by cellular growth and lower diffusion through the dense collagen, we decided to find out how much further damage a higher concentration of the photosensitiser could exert. Therefore, PDT using TPPS_2a_ (1 μg/mL) and longer exposure to light, (5 min) was tested, which resulted in considerable destruction across all cell types, from the cancer mass, the invading cancer cells, and both stromal cell types (Figure 4A). A recent study by Kucinska et al. (2021) [10] examined the effect of zinc phthalocyanine (M2TG3) in 2D and 3D cultures of prostate cancer (LNCaP cells) under normoxic and hypoxic conditions. As expected, their results showed that M2TG3 was less cytotoxic in 3D cell culture models than in the 2D cell culture under both conditions. Furthermore, no cytotoxic effect was observed in 3D cultures under hypoxic conditions. Their findings were consistent with those in our previous study [11].

PDT and PCI in complex 3D cancer models consisting of uncompressed type 1 collagen have been explored in a few studies. For example, O’Rouke et al. (2017) [4] examined the responsiveness of neurons and glia to PCI treatment using the same porphyrin utilised in our study (TPPS_2a_) or a chlorin photosensitiser (TPCS_2a_) and chemotherapeutic drug (Bleomycin) compared to head and neck squamous cell carcinoma (PCI30 cells), aiming to discover means of minimising nerve toxicity post-PCI treatment. According to their results, the neural and mixed glial cells showed less sensitivity (lower cell death) than PCI30 cells to PCI treatment using TPCS_2a_ or TPPS_2a_ which suggested that neurons are more resistant to PCI treatment than PCI30 cells and are able to survive in the conditions required for killing the tumour cells. In a similar study by Wright et al. (2009) [23] the same 3D culture system was employed to investigate the sensitivity of neurons and glia to PDT using the photosensitiser meta-tetrahydroxyphenyl chlorin (mTHPC), compared to breast carcinoma (MCF-7 cells); the model reflected the proximity of some tumours to the nervous system. Here, they observed that the cell viabilities were reduced by 48% in MCF-7 cells, 39% in glial cells and 11.9% in neurons when mTHPC was used at a concentration of 4 μg/mL, suggesting that the neurons showed significantly more resistance towards mTHPC-PDT than MCF-7 and glial cells.

Rizvi et al. (2013) [24] developed a 3D co-culture model (endothelialised ovarian micronodules) consisting of ovarian cancer (OVAR-5 cells) and HUVECs using the low adherence plate system. The OVCAR-5/HUVEC co-culture was then transferred into a GFR matrigel-coated plate to examine the potential of this model for testing PDT-mediated combination treatment. This study reported that the size of the OvCa spheroids decreased as the cell density of HUVECs increased which suggested some competition and inhibitory interactions between the two cell types. However, in our study, even though cancer and stromal cells were originally grown within specific compartments, the cancer cells were able to effectively invade far into the stroma regardless of the presence or absence of stromal cells and without any obvious effect on the size of cancer masses invading the stroma.

In our tumouroids, the HEY cancer cells were found to regrow and invade the stroma 7 days post-PDT treatment using TPPS_2a_ (0.5 μg/mL) and 3 min of exposure to light (Figure 7B and Figure 8). According to the in vivo study by Schumann et al. (2015) [25] a single dose of combination therapy with the photosensitiser phthalocyanine (Pc) and siRNA as a DJ-1 gene suppressor led to a complete eradication of ovarian tumours (grown from A2780/AD cancer cells) without any evidence of recurrence in mice, whereas PDT only treated tumours began to regrow 16 days after treatment. They further explained that the ROS and heat generated after a single dose of PDT could initially produce a strong response in the tumours. They hypothesised that the further combination of the treatment with DJ-1 suppression resulted in a superior therapeutic effect, thus preventing tumour regrowth. In our in vitro study, we observed that a short time post-PDT treatment (48 h), cells within the central cancer mass diminished and cells invading the stroma were also reduced. However, incubation for an additional 5 days resulted in the regrowth of the cancer cells. This could suggest that a single dose of PDT can provide a strong response to begin with, but the cancer cells are eventually able to overcome the conditions produced by PDT as the treatment loses the effect if the additional cycles of the treatment are not provided.

Since the oxygenation levels are likely to be higher at the edge of the cancer mass adjacent to the stroma than at the centre [18], we can postulate that PDT and PCI may elicit differential damage to the cells that invade the stroma compared to cells near the hypoxic core, but this would need further investigations.

## 5. Conclusions

In this study we explored PCI efficacy in complex in vitro 3D tumouroids constructed using human ovarian cancer cells as a central mass and fibroblasts and endothelial cells as a surrounding stroma, using TPPS_2a_ porphyrin as a photosensitiser and Saporin as a model cytotoxin.

PCI was shown to be effective in tumouroids, causing damage to the central cancer mass and cells that had invaded the stroma, as well as the stroma-resident non-cancer cells. Using higher photosensitiser and light doses, PDT caused lethal damage to all three cell types within the complex tumouroids. A lower dose PDT treatment used as a comparison in the PCI experiment was shown to cause damage to both the central cancer mass as well as in the stroma for up to 2 days after light illumination. However, in a separate experiment, it was found that tumouroids that had undergone the same PDT treatment showed complete regrowth 7 days after exposure to light. Such an effect has also been observed in other PDT studies [25]. Overall, the results of our study are promising since our 3D tumouroid has the capability to better mimic in vivo tumour models and can be used for further cancer therapy studies, delineation of mechanisms for different cell types and the modelling of treatment regimens for full cancer tissue destruction.

## Data Availability

Not applicable.
